# Light Transmission Aggregometry Versus VerifyNow for Antiplatelet Monitoring in Flow Diversion: A Retrospective Comparative Study

**DOI:** 10.3390/diagnostics16142155

**Published:** 2026-07-09

**Authors:** Andrey Petrov, Sergei Ermakov, Alexey Kornev, Oleg Belokon, Ruslan Sharshebaev, Vladimir Eliseev, Natalia Dryagina, Arkady Ivanov, Anna Petrova, Konstantin Samochernykh, Larisa Rozhchenko

**Affiliations:** 1 Vascular Neurosurgery Department, Polenov Neurosurgical Research Institute, Branch of Almazov National Medical Research Centre, 191014 Saint Petersburg, Russia; s.v.yermakov@yandex.ru (S.E.); nvdryagina@mail.ru (N.D.); .; petrovaanna2803@gmail.com (A.P.); neurobaby12@gmail.com (K.S.); rozhch@mail.ru (L.R.); 2 Belostrov Clinic of High Technologies, Clinic Beloostrov, 1, Yukki Urban Settlement, Vsevolozhsk District, Leningrad Region, 188651 Saint Petersburg, Russia; 3 «Vedanta» Medical Center, International School of Medicine, International University of Kyrgyzstan, Bishkek 720054, Kyrgyzstan; ruslansharshebaev@gmail.com; 4 Stavropol Krai State Budgetary Healthcare Institution “Stavropol Krai Clinical Hospital”, Semashko St., 1, 355030 Stavropol, Russia; alexeikornev@yandex.ru (A.K.); belokonoleg@gmail.com (O.B.); vve87@mail.ru (V.E.)

**Keywords:** flow diversion stents, intracranial aneurysms, antiplatelet therapy, light transmission aggregometry, VerifyNow, thromboembolic complications

## Abstract

**Background:** Over the past two decades, the widespread use of flow diversion stents (FDS) has significantly expanded treatment options for patients with intracranial aneurysms (IA). However, FDS implementation requires antiplatelet therapy (APT), the optimal regimen for which remains undefined. This study aimed to evaluate light transmission aggregometry (LTA) as a routine tool for monitoring antiplatelet therapy during flow diversion to examine whether an LTA-based residual-reactivity cut-off is associated with thromboembolic complications and to assess the agreement between LTA and VerifyNow (VN) P2Y12 reaction units (PRU), rather than to establish definitive safety thresholds. **Methods**: A retrospective analysis was conducted across two expert centers on 771 patients. Based on selection criteria, 203 IA patients who underwent FDS implantation between 2019 and 2023 with LTA-guided APT (clopidogrel plus aspirin) were included; LTA was used for monitoring in all 203 patients, and a subset of 84 patients additionally underwent VerifyNow testing for method comparison. The primary outcome was clinically significant thromboembolic complications (TECs) leading to a reduction in quality of life by ≥1 point on the modified Rankin scale (mRS). The secondary outcome was intracranial hemorrhage. **Results**: Among the 203 patients (203 FDS implantations), dual aggregometry control (LTA + VN) was performed in 84 cases. At the 12-month follow-up, complete or near-complete aneurysm occlusion was radiologically confirmed in 83.7% of patients. A favorable functional outcome (mRS ≤ 2) at 12 months was observed in 97.5% of cases. Thromboembolic complications occurred in 7 patients (3.4%) and hemorrhagic complications in 4 (2.0%). On receiver operating characteristic (ROC) analysis, the control-LTA cut-off of 44% showed only weak, statistically non-significant discrimination for thromboembolic events (area under the curve, AUC 0.700, 95% confidence interval, CI 0.479–0.920; *p* = 0.073). In the 84 patients tested with both assays, LTA and VerifyNow PRU correlated only moderately (Spearman’s ρ = 0.51; Pearson’s r = 0.53) and showed wide Bland–Altman limits of agreement, indicating that the two methods are not clinically interchangeable. **Conclusions**: Routine platelet-function aggregometry may be a useful tool for monitoring APT during flow diversion. In this retrospective cohort, lower residual platelet reactivity on LTA was associated with fewer thromboembolic and hemorrhagic events; however, given the small number of outcome events (*n* = 7) and an LTA cut-off whose discriminative ability did not reach statistical significance, this association is exploratory and hypothesis-generating rather than an established safety threshold. LTA and VerifyNow PRU were not clinically interchangeable, so no VerifyNow cut-off is proposed. Prospective, adequately powered studies with outcomes stratified directly by each assay are required to define and validate clinically applicable thresholds.

## 1. Introduction

The active introduction of flow diversion stents (FDS) into clinical practice for treating patients with intracranial aneurysms (IA) at the initial stages was accompanied by a significant number of thromboembolic complications (TECs) [1]. Since then, devices have undergone significant design changes, but their metal surface area is still 3–5 times larger than that of intracranial stents [2]. Despite this, thanks to modern coatings and materials, FDS can have improved antithrombotic properties [3,4]. Moreover, an increased surface area can contribute to better endothelization of the stent and improve its biocompatibility, reducing the risk of complications [5].

However, these arguments do not negate the need for antiplatelet therapy (APT) in treatment with FDS. An individual approach to choosing APT can compensate for potential risks associated with device features [6].

The most common APT regimen is considered to be a combination of clopidogrel and acetylsalicylic acid. However, to date, significant data has been collected on resistance to this regimen, reaching 40% of the population. Also, there is varying effectiveness of antiplatelet drugs available on the market [7,8,9,10]. Assessment of platelet aggregation inhibition plays an important role in modern medicine. Especially in the context of intracranial aneurysm treatment, light transmission aggregometry has traditionally been regarded as the ‘gold standard’ for platelet-function testing, although it remains poorly standardized across laboratories [11,12,13,14]. Despite the widespread use of light transmission aggregometers for more than half a century [11,12], a single algorithm has not been developed for their use in patients undergoing endovascular aneurysm treatments.

The lack of established reference values for light transmission aggregometry (LTA) in endovascular neurosurgery creates difficulties in interpreting results. The Society of NeuroInterventional Surgery (SNIS) notes significant variability in preoperative platelet function tests in different neurointervention centers, which prevents the establishment of a clear diagnostic range for LTA. This highlights the need for research and standardization to increase the effectiveness of the technique in clinical practice [15].

The aim of this study was to evaluate LTA as a routine tool for monitoring antiplatelet therapy in flow diversion, to examine whether an LTA-based residual-reactivity cut-off is associated with thromboembolic complications, and to assess the agreement between LTA and VerifyNow P2Y12 reaction units (PRU) rather than to establish a definitive safety threshold.

## 2. Materials and Methods

### 2.1. General Characteristics of Patients

Our paper presents a retrospective analysis of the use of LTA for preoperative platelet function testing in patients with IA treated with FDS. A retrospective study was performed based on hospital registries of patients with intracranial aneurysms from two clinical centers with more than 5 years of experience using LTA in routine practice (Polenov Neurosurgical Research Institute, St. Petersburg, Russia; Stavropol Regional Clinical Hospital, Stavropole, Russia). Considering the requirements for experience in performing LTA, we analyzed the results of treating patients from 2019 to 2023 according to the following criteria: patients who received FDS and underwent follow-up clinical and angiographic examinations within a period of 6–12 months after surgery.

The study included 203 out of 771 patients. The average age was 52.9 ± 12.6 (95% CI: 51.1–54.6). Among the included patients, women predominated: 170 (83.7%).

### 2.2. Inclusion and Exclusion Criteria

The inclusion criteria included FDS implantation of a similar design (pipeline embolization device Flex (PED Flex) (Medtronic, Irvine, CA, USA), Derivo (Acandis, Pforzheim, Germany), SILK-Plus (BALT, Montmorency, France), with preoperative assessment of the effectiveness of APT using the LTA method, clinical evaluation, and angiographic monitoring after 6–12 months.

**Figure 1:**
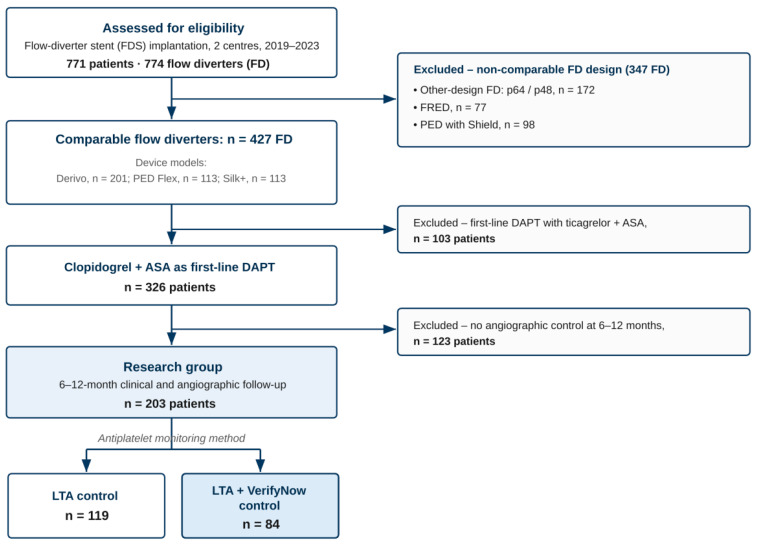
Flow diagram of device and patient selection. Across two centers (2019–2023), 771 patients received 774 flow diverters (FD) for intracranial aneurysm under LTA-guided antiplatelet therapy. After exclusion of non-comparable device designs (347 FD: p64/p48, *n* = 172; FRED, *n* = 77; PED with Shield, *n* = 98), 427 comparable FD remained (Derivo, *n* = 201; PED Flex, *n* = 113; Silk+, *n* = 113). At the patient level, 103 patients receiving ticagrelor + acetylsalicylic acid (ASA) as first-line dual antiplatelet therapy (DAPT) were excluded; 326 patients received clopidogrel + ASA as first-line DAPT. After excluding 123 patients without protocol-mandated 6–12-month clinical and angiographic follow-up, 203 patients formed the research group, all monitored by light transmission aggregometry (LTA); of these, 119 were monitored by LTA alone and 84 by LTA plus VerifyNow. The device-level count (FD) and the patient-level counts are reported separately because some patients received more than one comparable flow diverter (ASA, acetylsalicylic acid; DAPT, dual antiplatelet therapy; FD, flow diverter; LTA, light trans-mission aggregometry). Box shading marks the two analysed groups: the light-blue box denotes the final research group (*n* = 203) and the darker-blue box the LTA + VerifyNow dual-monitoring subgroup (*n* = 84); all other (unshaded) boxes represent enrolment, selection and exclusion steps.

The exclusion criteria included acute period of aneurysmal hemorrhage, any intracranial stent implantation in the anamnesis, allergy to thienopyridine blockers P2Y12 or acetylsalicylic acid, gastric ulcer in acute stage, thrombocytopenia (PLT < 100 × 10^9^/L), somatic diseases in decompensated stage (congestive heart failure, lung disease, liver disease, kidney disease), oncological diseases, and pregnancy or lactation. The patient selection process is illustrated in Figure 1.

Detailed baseline data for the 568 excluded patients were not uniformly available in the registries, as most were excluded for absence of protocol-mandated 6–12-month angiographic follow-up rather than for clinical reasons; a formal included-vs-excluded comparison was therefore not feasible. This is acknowledged as a source of potential selection bias (see Limitations).

### 2.3. Antiplatelet Therapy and Assessment of Its Effectiveness

The pre-analytical stage of light transmission aggregometry (LTA) was carried out in strict accordance with the current guidelines of the International Society on Thrombosis and Hemostasis (ISTH). Venous blood was collected by puncturing the cubital vein using needles with a gauge of at least 21 G into plastic tubes containing a 3.2% (0.109 M) sodium citrate solution in a 9:1 ratio. In order to circumvent the occurrence of artifactual platelet activation, it was imperative to avert prolonged venous stasis. Consequently, the initial 2–3 milliliters of blood were discarded. In order to obtain platelet-rich plasma (PRP), whole blood was subjected to centrifugation at an angular velocity ranging from 150 to 200× *g* for a duration of 10 to 15 min at ambient temperature. It is imperative to note that the use of the centrifuge brake was excluded during this process. Platelet-poor plasma (PPP), which is necessary to establish a baseline of 100% light transmission, was obtained by centrifuging the remaining volume of blood at speeds ranging from 2000 to 2500× *g* for a duration of 15 to 20 min. It is imperative to note that all samples were stored exclusively at ambient temperature (20–25 °C). This precautionary measure was implemented to avert the potential for spontaneous platelet activation, which might be triggered by cooling. Aggregometry was performed within a validated time window, specifically between 1 and 4 h after venipuncture. Prior to the addition of the inducer (adenosine diphosphate, ADP 5 µmol/L), the cuvettes were subjected to an incubation process at a temperature of 37 °C, with a magnetic stirrer speed of 1000 rpm.

All patients underwent an evaluation of platelet aggregation before the initiation of APT, after which clopidogrel 75 mg/day plus acetylsalicylic acid 100 mg/day was prescribed. On the sixth day of administration, LTA was performed to monitor APT efficacy using an ALAT-2 manual laser platelet analyzer (Biola, Moscow, Russia) and a VerifyNow P2Y12 analyzer (Werfen, L’Hospitalet de Llobregat, Spain). LTA was performed in platelet-rich plasma with ADP as the inducer at a final concentration of 5 µmol/L.

The effect of clopidogrel was found to be effective in reducing platelet activity by less than 50%. The choice of the upper limit for reducing optical density was based on research data and our own experience with the LTA technique, as well as the use of another P2Y12 receptor blocker, ticagrelor. Therapy lasted from 6 to 12 months. To monitor the effectiveness of antiplatelet treatment, 84 patients had platelet function assessed using the VerifyNow P2Y12 analyzer, which measures adenosine diphosphate-induced platelet aggregation by an increase in light transmission and uses a proprietary algorithm to calculate PRU values (P2Y12 reaction units).

Both centers used the same ALAT-2 platelet analyzer model and a shared standardized operating protocol. Instruments were calibrated against platelet-poor plasma (set as 0% aggregation) and platelet-rich plasma (100%) before each session, and measurements were performed by trained operators following identical pre-analytical and analytical conditions to minimize inter-center and inter-operator variability.

### 2.4. Endovascular Treatment

Surgical treatment was performed under general anesthesia using Philips Allura B20 and Azurion 7 M20 angiographic systems (Philips, Amsterdam, The Netherlands), with transfemoral access. A 6 or 7 Fr Hyde introducer was inserted into the cervical segment of the internal carotid or vertebral artery. The size and morphometric characteristics of the aneurysm were determined using selective cerebral 3D rotational angiography and a 0.027 “microcatheter and 0.14 “microwire were used for FDS placement. FDS were selected for IA occlusion including Pipeline Embolization Device (PED) Flex from Medtronic (Irvine, CA, USA), Derivo from Acandis (Germany) and SILK-Plus from BALT (France). The success of aneurysm closure was assessed immediately after FDS insertion and at 6–12 months using the Raymond-Roy scales and Cekirge–Saatci scales.

### 2.5. Review of Medical Records

In this study, a thorough analysis of medical records was conducted to assess the effectiveness of treatment and identify potential complications. All key patient characteristics were recorded, including demographic data (age, gender, presence of comorbidities such as hypertension, diabetes mellitus, atrial fibrillation, dyslipidemia, etc.), medical history, information about previous treatments, as well as imaging data including angiography, computed tomography (CT), and magnetic resonance imaging (MRI), depending on the patient’s clinical situation.

### 2.6. Endpoints

The primary endpoint was the occurrence of clinically significant thromboembolic events (a decrease in quality of life by 1 point or more on the modified Rankin scale (mRS)) within 12 months after surgery. The secondary endpoint was intracranial hemorrhagic events within 1 year.

### 2.7. Data Analysis

Statistical data analysis was carried out using the software StatTech v. 4.14.3 (developed by—StatTech LLC, Moscow, Russia) and jamovi 2.7.34 with the General Analyses for Linear Models (GAMLj) v. 2.6.6 package. Descriptive statistics, between-group comparisons, and correlation and regression analyses were performed using StatTech v. 4.14.3: categorical variables are presented as absolute and relative frequencies (%) with 95% confidence intervals (CI); quantitative variables with a non-normal distribution are presented as the median (Me) and interquartile range (Q_1_–Q_3_). The Mann–Whitney U test was used to compare quantitative measures between two independent groups; for categorical variables, Fisher’s exact test was used due to the low absolute frequencies of events in individual cells of the contingency tables. Agreement between control LTA and VerifyNow PRU was evaluated with Spearman’s and Pearson’s correlation and with Bland–Altman analysis. A simple linear regression of PRU on LTA was used solely as an exploratory calibration step underlying the Bland–Altman comparison (to convert LTA values to expected PRU); it was not used to derive a clinical VerifyNow threshold, and the regression equation and its plot are therefore not reported as a result. The discriminatory ability of the control LTA in predicting thromboembolic complications (TECs) was assessed by constructing a receiver operating characteristic (ROC) curve with calculation of the area under the curve (AUC) and its 95% CI; the optimal cut-off level was determined by maximizing the Youden index. Firth’s penalized likelihood logistic regression—for both univariate and multivariate analysis of potential predictors of TEC—was performed using the jamovi 2.7.34 software package with the GAMLj v. 2.6.6 add-on; odds ratios (OR) and their 95% confidence intervals were calculated for all variables. A *p*-value of <0.05 was adopted as the critical level of statistical significance in all types of analysis.

## 3. Results

### 3.1. Study Population and Baseline Characteristics

From 2019 to 2023, 771 patients underwent FDS (774) implantations in our centers under the supervision of LTA. Of these, 203 met the inclusion criteria. The average follow-up period was 10.00 ± 2.6 months (95% CI 9.6–10.4).

In 84 patients included in the analysis, residual platelet activity was determined using the VerifyNow aggregometer in parallel with the LTA control to construct a cross-validation model. The baseline characteristics of the patients are presented in Table 1.

### 3.2. Baseline Platelet Reactivity and Antiplatelet-Therapy Adjustment

All patients underwent an assessment of platelet activity using LTA. After this, they were treated with clopidogrel (75 mg) and ASA (100 mg) once a day for 6 days. The median value of baseline LTA was 73% (Q1–Q3: 65–80). Anamnestic data were taken into account, as well as assessments of cardiovascular risk factors and non-steroidal anti-inflammatory drugs (NSAIDs) and novel oral anticoagulants (NOA) use (Table 2).

NSAIDs intake significantly influences the level of baseline LTA. Vascular risk factors and NOA intake have no effect on initial platelet aggregation. Following a six-day treatment period with clopidogrel and ASA, a subsequent LTA revealed a reduction in aggregation of less than 50% in 48 patients (23.6%), indicating inadequate responsiveness to clopidogrel. All these patients were switched from clopidogrel to ticagrelor 90 mg × 2/day. Importantly, this ticagrelor group (*n* = 48) consisted of clopidogrel non-responders who were switched to ticagrelor after an inadequate on-treatment response; it is distinct from the 103 patients who received ticagrelor + ASA as first-line DAPT and were excluded from the study (Figure 1). The repeat LTA, which served as the foundation for the decision to transition to ticagrelor, was assessed while patients were on clopidogrel. Subsequent LTA measurements following the transition to ticagrelor were not part of the standard of care for all patients in this subgroup.

### 3.3. Clinical Outcomes

Thromboembolic complications (TECs) developed in seven patients (3.4%) during LTA monitoring with a threshold of 50%. An increase in mRS of ≥1 point was observed in each case. Hemorrhagic complications were confirmed in four patients (2%). Treatment outcomes are presented in Table 3.

A notable limitation is that the transition to ticagrelor was not arbitrary or protocol-based, but rather, it was a selective decision made only in patients with laboratory-confirmed inadequate responsiveness to clopidogrel. Therefore, the “ticagrelor” group was, by definition, comprised of patients with elevated baseline residual platelet reactivity in comparison to those who continued on clopidogrel. Consequently, the comparison of the incidence of thromboembolic and hemorrhagic complications between the two antiplatelet therapy groups reflects not only the pharmacodynamic differences between the drugs but also the baseline differences between the patient populations being compared.

The confirmation of all complications was achieved through the implementation of neuroimaging studies, which included multi-detector CT and/or magnetic resonance imaging. Of the seven thromboembolic events, four occurred in patients who had been switched to ticagrelor (4/48, 8.3%), and three occurred in patients who had continued to take clopidogrel (3/155, 1.9%). In the ticagrelor group, TEC was associated with medication errors in two patients (1.0% of the entire cohort, 4.2% of the ticagrelor group) and with technical difficulties requiring balloon dilatation in one patient (0.5% and 2.1%, respectively), respectively. In one case, the cause remained unclear. In the clopidogrel group, TEOs were attributed to technical difficulties during stent implantation in two patients (1.0% of the entire cohort, 1.3% in the clopidogrel group) and to stent occlusion in the internal carotid artery in one patient (0.5% and 0.6%). In one case, TEO resulted in a fatal ischemic stroke, documented on day 67 after surgery (0.5%), in a patient who had voluntarily discontinued antiplatelet therapy.

Hemorrhagic complications were more frequent with ticagrelor plus ASA than with clopidogrel plus ASA (Fisher’s exact test, *p* = 0.042). All four hemorrhagic events were fatal: three of 48 patients in the ticagrelor group (6.2%) and one of 155 in the clopidogrel group (0.6%). This comparison should be interpreted with caution because the ticagrelor group was, by indication, enriched for patients with higher baseline residual reactivity and is not directly comparable to the clopidogrel group.

### 3.4. Residual Platelet Reactivity and Thromboembolic Events

**Figure 2:**
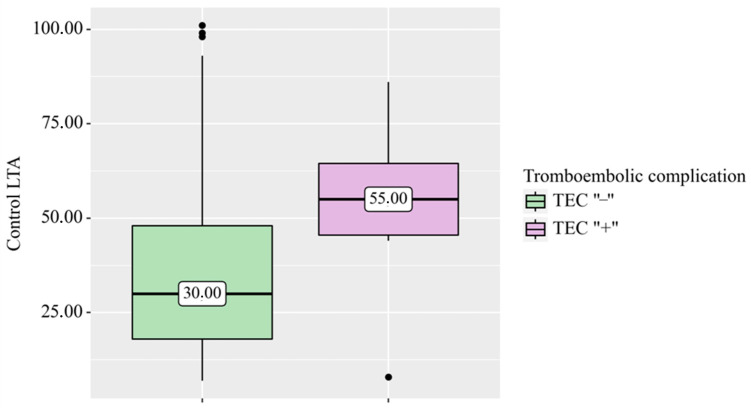
Distribution of control LTA values in patients with versus without thromboembolic complications (TECs). The box plot shows the median, interquartile range and full range; *p* = 0.073, Mann–Whitney U test. In the legend, TEC “−” denotes patients without and TEC “+” patients with thromboembolic com-plications.

The mean baseline LTA at an ADP concentration of 5 µmol/L was 73.1 ± 14.4%. On the sixth day of clopidogrel treatment, the mean residual aggregation activity was 37.9 ± 23.9%. As demonstrated in Figure 2, patients with clinically significant complications exhibited higher levels of residual LTA compared to those without such complications. However, this difference did not reach statistical significance (*p* = 0.073, Mann–Whitney U test).

The median control LTA was 30% in patients without TEC and 55% in patients with TEO, with a wider interquartile range observed in the group with complications. In four patients with TEC, all of whom were part of the ticagrelor group, the median residual LTA exceeded the 50% safety threshold, accounting for 1.97% of the total cohort. In three patients with TEC (1.48% of the cohort), LTA was ≤50%, indicating that TEC was not caused solely by high residual platelet reactivity.

### 3.5. Discriminative Performance of Control LTA (ROC Analysis)

**Figure 3:**
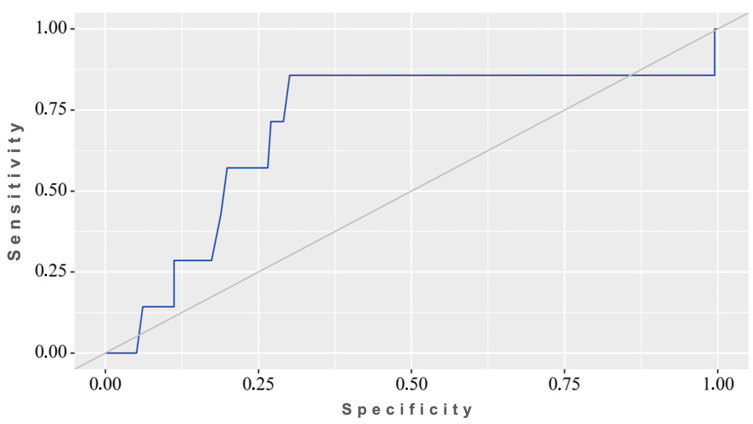
ROC curve for control LTA predicting thromboembolic complications (AUC = 0.700, 95% CI 0.479–0.920; *p* = 0.073). Youden-optimal cut-off, 44% (sensitivity 85.7%, specificity 69.9%). The non-significant AUC, with a 95% CI crossing 0.5, indicates weak, statistically unconfirmed discrimination; the threshold is hypothesis-generating (203 patients, 7 events).

ROC analysis of the control LTA for predicting TEC yielded an AUC of 0.700 (95% CI 0.479–0.920, *p* = 0.073), indicating weak and statistically insignificant discriminatory power. The cut-off point that maximized the Youden index (0.56) was determined to be 44%. The sensitivity was found to be 85.7%, while the specificity was 69.9%, as illustrated in Figure 3.

Given that the lower limit of the 95% confidence interval for the area under the curve (AUC) (0.479) is less than 0.5, which is equivalent to random guessing, and the model itself did not attain statistical significance (*p* = 0.073), the discriminatory capability of the control LTA in predicting TEC remains unvalidated from a statistical standpoint. Consequently, the 44% cut-off calculated using the Youden index represents a hypothesis-generating estimate that is research-based, and it cannot be recommended as a clinical decision-making cut-off until it has been validated in an independent and larger sample.

### 3.6. Agreement Between LTA and VerifyNow PRU

In the 84 patients assessed with both methods, control LTA showed a statistically significant but only moderate positive correlation with VerifyNow PRU (Spearman’s ρ = 0.51; Pearson’s r = 0.53; both *p* < 0.001). Because the two assays are reported on different scales and the correlation explained only part of the variance, a correlation of this strength does not establish that the methods are interchangeable; accordingly, no regression equation is presented, and no VerifyNow threshold is derived.

A Bland–Altman analysis was performed to assess the agreement between the two methods for determining residual platelet reactivity: the actual VerifyNow PRU values measured were compared with the calculated PRU values obtained by converting the LTA parameters. The mean systematic bias was ≈0 PRU, indicating the absence of systematic error in the calibration model for this sample. However, the 95% confidence limits proved to be wide: from −94.9 to +94.9 PRU. Given that the clinically significant resistance thresholds on the VerifyNow scale lie within the range of 180–240 PRU, the width of the confidence intervals—comparable to half of this range—indicates a lack of clinically acceptable interchangeability between the LTA and VerifyNow PRU methods in individual measurements, despite a statistically significant moderate correlation between them at the group level.

### 3.7. Predictors of Thromboembolic Complications: Logistic Regression

A formal assessment of model calibration (the standard Hosmer–Lemeshow test with a 10-group split) was not carried out. As with an extremely small number of outcomes (*n* = 7), such a split results in fewer than one event per group, rendering the test statistics uninterpretable. As an alternative, a simplified comparison was carried out between the observed and expected frequencies of TECs in three risk groups (based on tertiles of predicted probability): no statistically significant differences were identified (χ^2^ ≈ 2.76; *p* = 0.097); however, given the low power of this test, the result cannot be interpreted as confirmation of adequate model calibration.

The results of a univariate logistic regression analysis of the association between potential predictors and the development of thromboembolic complications are presented in Table 4.

Due to the limited number of outcomes (*n* = 7), logistic regression with Firt’s correction was employed to obtain robust estimates. Odds ratios (OR) and 95% confidence intervals are reported for all variables.

In the regression analysis, variables for which only one event was observed in any one category were excluded. The categories in question include coronary heart disease, a history of aneurysm cavity occlusion with microspirals, and balloon dilatation following FD implantation.

Statistically significant associations with the incidence of TEC were found for both the generation of the implanted stent and aneurysm localization. The use of first-generation stents was associated with a higher likelihood of complications (≈5.3-fold) compared with second-generation stents (OR 5.34; 95% CI 1.30–32.39; *p* = 0.035). After grouping all non-ICA sites as “Other”, aneurysms located above the ICA bifurcation or in the vertebrobasilar territory showed a significantly higher probability of TEC (≈5.9-fold; OR 5.85; 95% CI 1.25–27.04; *p* = 0.024). Exceeding the 50% threshold on control LTA was associated with a higher probability of TEC (≈4.4-fold; OR 4.41; 95% CI 0.94–23.74; *p* = 0.061), although this borderline estimate should be read in the context of the protocol-driven switch of antiplatelet therapy.

To assess the independent predictors of thromboembolic complications, selected on the basis of univariate screening, a multivariate logistic model with Firt’s correction was constructed, incorporating the generation of the flow-diverter stent, the location of the aneurysm, and residual platelet aggregation (LTA > 50%). The model proved to be statistically significant overall (*p* = 0.041). The adjusted odds ratio for second-generation stents was 0.35 (95% CI 0.07–1.78; *p* = 0.21) for aneurysms located above the ICA bifurcation or in the vertebrobasilar basin—4.12 (95% CI 0.74–22.9; *p* = 0.11) and for LTA > 50%—2.58 (95% CI 0.56–11.9; *p* = 0.22). It should be emphasized that, with a ratio of 7 events to 3 predictors (events per variable, EPV ≈ 2.3), the multivariate model is not statistically robust; despite the application of the Firth correction, the presented OR estimates and their confidence intervals should be interpreted with caution as preliminary findings requiring confirmation in a larger sample.

Thus, after simultaneously adjusting for all included factors, none of the predictors retained independent statistical significance, although an aneurysm located above the ICA bifurcation or in the vertebrobasilar basin showed a tendency towards a higher incidence of thromboembolic complications.

## 4. Discussion

The use of FDS is still associated with the prescription of antiplatelet therapy, the standards for monitoring and evaluating the effectiveness of which are not regulated in vascular neurosurgery. Despite significant progress in the safety of FDS, procedures related to its implantation require APT to reduce risk of complications. DAPT is most commonly used in neurosurgical practice and includes ASA and another anti-platelet agent, usually a thienopyridine derivative (clopidogrel, ticagrelor, or prasugrel), which has been well studied in cardiology but remains a subject of debate in vascular neurosurgery [16,17,18,19,20,21,22].

The question of the optimal APT regimen remains open. This is confirmed by the high indicator of reduced sensitivity to clopidogrel due to polymorphism of CYP2C19 [8,23,24]. Against this background, one possible solution to the problem could be routine administration of ticagrelor. However, it is extremely rare for patients to receive this drug [25,26].

Despite the growing popularity of experts’ opinion on choosing ticagelor as a first-line treatment, it is important to pay attention to its pharmacokinetic features. In our cohort, the ticagrelor + ASA combination was associated with more thrombo-embolic complications after discharge, possibly related to missed doses given its short (~7 h) half-life; this comparison is, however, confounded by indication, as ticagrelor-treated patients had higher baseline residual reactivity [27,28].

Another thienopyridine derivative is prasugrel, which has pharmacological advantages over clopidogrel as it is converted more efficiently into an active metabolite. However, the more potent effect of prasugrel increases the risk of bleeding, which must be taken into account when choosing a drug [29,30].

Thus, given the characteristics of anti-platelet drugs, platelet-function testing before intracranial stent implantation appears increasingly justified, although its routine clinical benefit remains to be confirmed, even considering the low incidence of patients being insensitive to ticagrelor or prasugrel. Nevertheless, it is important to select an appropriate method for assessing platelet function.

Molecular genetic methods are becoming popular for detecting platelet dysfunction, but they are not a priority for evaluating the effectiveness of APT [31,32]. Viscoelastic methods, such as thromboelastography, rotational thrombelastometry, clotpro, and ultrasound sonoremetry, more accurately determine the efficacy of APT and provide a complete picture of the clotting process in real-time, including clot formation, stabilization, and fibrinolysis. However, these methods are limited by their high cost and difficulty in interpreting results. Standardizing and validating these methods for assessing the efficacy of APT is still being developed and raises questions about their reliability in different clinical situations [2,33,34,35,36].

These limitations support the continued use of LTA—a widely used reference method—for detecting both hereditary and acquired qualitative platelet-function disorders. LTA is an accessible and well-studied method for assessing platelet function in patients with IA. Despite extensive experience with its use, there are no uniform standards or protocols in vascular neurosurgery, making it difficult to compare results between medical institutions and research groups [15,37].

It should be acknowledged that the clinical value of routine platelet-function testing remains debated. Brinjikji et al. [21] reported that platelet testing before Pipeline implantation was associated with worse clinical outcomes, likely reflecting selection of higher-risk patients and heterogeneous response-adjustment strategies rather than harm from testing itself. Our findings should therefore be interpreted as supporting standardized, protocol-driven monitoring, rather than testing per se, and underline the need for prospective validation of any reactivity threshold.

One of the most challenging aspects in applying various methods for measuring platelet aggregation using LTA is the interpretation of results, which in turn is linked to the complexity of the technique. This is partially due to significant variability in LTA performance associated with the use of different coagulation activator concentrations and the influence of numerous preanalytical factors, such as needle diameter, venous blood sampling techniques, and delivery time to laboratory. However, adherence to standardized protocols, regular calibration of equipment, and conducting studies with controls can minimize their impact. It is also crucial to consider individual patient characteristics, such as medication intake and concomitant conditions, which may affect test results [13,14].

In a study by Issei Kan [2], an LTA threshold value of 60% was established at which the AAT regimen was changed. The proposed threshold was found to be safe for implantation of assistive stents. For FDS in the work of Nimer Adeeb [38], which included 402 patients, a light transmission of 50% (ADP at a concentration of 5 mmol/L) was determined as the upper limit of LTA. Safety of the proposed reference value was supported by a lower frequency of TEC (1.3% in patients with LTA < 50 vs. 20.0% in those with LTA > 50).

In our study, the upper threshold for residual platelet activity was set at 44% light transmission at LTA. Similar values were used in the work of Alejandro Enriquez-Marulanda [39]. An analysis of the treatment of 442 patients using FDS and monitoring the effectiveness of DAPT, using the LTA method as in our study, showed a decrease in the frequency of TEC to 4.9% and HCT to 1.9%. The typical ranges are demonstrated by Yang Yang Zhou, who confirmed the pattern described above. For assisting stents, the upper limit of LTA was higher than that for FDS and amounted to 42.9%, whereas for FDS it was 36.4% respectively [7]. However, ADP concentrations of 2.5 and 10 μmol/L were used, making it difficult to compare and interpret the results. Nevertheless, in a group of patients with decreased platelet aggregation capacity below target values, more than a 5-fold reduction in TEC was found. Despite the considerable experience gained in using LTA for diagnosing hemostatic system pathologies and monitoring the effectiveness of antiplatelet therapy, reference values for endovascular neurosurgical procedures have not yet been established. Furthermore, a significant number of preanalytical factors that affect the accuracy of LTA contributed to the development of the VerifyNow automated aggregometry system, which proved to be a fast, efficient, and easy-to-use technique. However, despite its advantages, there are still significant differences in interpreting the results of VerifyNow. In early studies [40,41,42,43], the upper safety limit was set at 205–230 PRUs. In later studies, there has been a tendency to lower the upper safety threshold when performing VerifyNow to monitor the effectiveness of antithrombotic therapy during femoral artery stenting [9,44].

Subsequent studies had shown a downward trend in this threshold. In prospective studies of COMATS 1 and COMAT2, a new algorithm for interpreting PRU VN [5,45] data was proposed: more than 240 showed no response to antiplatelet therapy, 100–239 showed reduced response, and less than 100 showed sufficient platelet inhibition by P2Y2 receptor blockers. The use of this grading was associated with a reduced risk of thromboembolic complications during endovascular procedures.

Summarizing the above, standardized methods for assessing platelet function will allow for large-scale multicenter studies. These studies will lead to an accumulation of more reliable data on how various factors affect platelet function in patients with IA. This could contribute to the development of personalized treatment approaches and improved long-term outcomes for endovascular interventions.

However, there are potential limitations to this study. The majority of patients included in the study were Caucasian. Patients in the acute phase of IA rupture were excluded, so the results may not be applicable to these patients. Additionally, the study was conducted at two centers, which could affect the accuracy of the LTA technique used in the aggregometry procedure despite efforts to standardize it.

The following limitation should be noted: Due to the limited number of thromboembolic events (*n* = 7), the study’s statistical power to detect effects of moderate magnitude is limited. Additionally, the cut-off values derived from ROC and logistic regression analyses are susceptible to the risk of overfitting and instability in the estimates. The thresholds and odds ratios presented herein are regarded as preliminary hypotheses that require confirmation in independent and larger samples. These results should be generalized with caution to populations with different characteristics (e.g., other types of flow-diverter devices or antiplatelet therapy regimens). A notable constraint of this investigation is the possibility of selection bias. Of the initial cohort of 771 patients who underwent FDS implantation, only 203 patients (26.3%) were included in the final analysis. The exclusion of nearly three-quarters of the initial population was dictated by strict selection criteria, in particular the requirement for angiographic follow-up results at 6–12 months. Patients who did not attend their scheduled follow-up examination may have been divided into two distinct groups: those with excellent clinical outcomes who lacked motivation to attend and, conversely, those with severe adverse events that prevented them from undergoing follow-up angiography. Consequently, the incidence of thromboembolic (3.4%) and hemorrhagic (2.0%) complications documented in our sample might not accurately reflect the true prevalence in general clinical practice. In order to eliminate this bias and accurately validate the proposed cut-off values, prospective studies with strict control and minimization of loss to follow-up are required.

## 5. Conclusions

Routine platelet-function aggregometry may be a useful tool for monitoring APT during flow diversion. In this retrospective cohort, lower residual platelet reactivity on LTA was associated with fewer thromboembolic and hemorrhagic events; however, given the small number of outcome events (*n* = 7) and an LTA cut-off whose discriminative ability did not reach statistical significance (AUC 0.700, 95% CI 0.479–0.920; *p* = 0.073), this association is exploratory and hypothesis-generating rather than an established safety threshold. In this sample, LTA and VerifyNow PRU were not clinically interchangeable: their correlation was modest and the Bland–Altman limits of agreement were too wide for individual-patient calibration, so no VerifyNow cut-off is proposed. Prospective, adequately powered studies with outcomes stratified directly by each assay are required to define and validate clinically applicable thresholds.

## Figures and Tables

**Table 1:** Descriptive statistics for patients.

Variables		Categories		Abs.		%			95% CI
**Categorical Variables**									
Groups		Group 1		119		58.6			51.5–65.5
		Group 2		84		41.4			34.5–48.5
APT control		LTA		119		58.6			51.5–65.5
		LTA + VN		84		41.4			34.5–48.5
Gender		M		33		16.3			11.5–22.1
		F		170		83.7			77.9–88.5
FDS type		DERIVO		86		42.4			35.5–49.5
		PED FLEX		55		27.1			21.1–33.8
		SILK+		62		30.5			24.3–37.4
Localization		ACoA		4		2.0			0.5–5.0
		BA		9		4.4			2.0–8.2
		ICA		164		80.8			74.7–86.0
		MCA		22		10.8			6.9–15.9
		PCA		1		0.5			0.0–2.7
		VA		3		1.5			0.3–4.3
Arterial hypertension				115		56.7			49.5–63.6
Diabetes mellitus				22		10.8			6.9–15.9
Smoking				46		22.7			17.1–29.0
Coronary heart disease				30		14.8			10.2–20.4
Coil occlusion				56		27.6			21.6–34.3
Balloon dilation				6		3.0			1.1–6.3
Multiple aneurysms				54		26.6			20.7–33.2
**Quantitative variables**									
**Variables**	**Me**		**Q_1_–Q_3_**		***n***		**Min**	**Max**	
Age	54.00		44.00–63.00		203		18.00	79.00	
mRs pre-op	0.00		0.00–1.00		203		0.00	3.00	
NIH pre-op	0.00		0.00–0.00		203		0.00	8.00	
Aneurysm width	6.00		4.00–10.55		203		2.00	38.00	
Aneurysm height	5.50		3.60–10.00		203		1.50	35.00	
Aneurysm neck width	5.00		3.05–6.20		203		2.00	42.00	

**Table 2:** Analysis of basic LTA conditioning on risk factors.

Variables	Categories	Basic LTA			*p*
		Me	Q_1_–Q_3_	*n*	
Arterial hypertension	no	73.50	66.50–81.25	88	0.695
	yes	72.00	65.00–80.00	115	
Diabetes mellitus	no	73.00	65.00–81.00	181	0.268
	yes	71.50	64.25–76.50	22	
Smoking	no	73.00	62.00–81.00	157	0.830
	yes	71.00	67.25–78.75	46	
Coronary heart disease	no	73.00	64.00–80.00	173	0.553
	yes	73.00	67.00–80.00	30	
Taking NSAIDs	no	74.00	66.00–81.00	187	0.011 *
	yes	65.00	60.50–71.25	16	
Atrial fibrillation	no	72.00	65.00–80.00	195	0.775
	yes	75.00	69.75–78.25	8	
Taking NOA	no	72.00	65.00–80.00	197	0.456
	yes	76.50	74.50–80.75	6	

*—differences are statistically significant (*p* < 0.05).

**Table 3:** Descriptive statistics for patient after treatment (50% LTA).

**Variables**	**Me**	**Q_1_–Q_3_**	***n***		**Min**		**Max**
mRs post-op	0.00	0.00–1.00	203		0.00		6.00
mRs control	0.00	0.00–0.00	203		0.00		6.00
NIH post-op	0.00	0.00–0.00	200		0.00		24.00
**Qualitative variables**							
**Variables**	**Categories**	**Abs.**		**%**		**95% CI**	
Thromboembolic complication	TEC “−”	196		96.6		93.0–98.6	
	TEC “+”	7		3.4		1.4–7.0	
Hemorrhagic complications	HC “−”	199		98.0		95.0–99.5	
	HC “+”	4		2.0		0.5–5.0	
Balloon dilation after FDS implantation	no	197		97.0		93.7–98.9	
	yes	6		3.0		1.1–6.3	

“−” and “+” indicate the absence and presence of the event, respectively (TEC, thromboembolic complications; HC, hemorrhagic complications).

**Table 4:** Univariate logistic regression of candidate predictors of thromboembolic complications (TEC; 203 patients, 7 events), estimated with Firth’s correction. Odds ratios (OR) are reported with 95% confidence intervals (CI) and *p*-values. “LTA > 50%” denotes residual platelet aggregation above 50% on control light transmission aggregometry.

Variables	Categories	*n*	TEC (*n* = 7)	OR (95% CI)	*p*-Value
Gender	F	170	5 (71.4%)	2.39 (0.43–16.76)	0.32
	M	33	2 (28.6%)		
Stent generation	FD 2 gen.	141	2 (28.6%)	5.34 (1.30–32.39)	0.035
	FD 1 gen.	62	5 (71.4%)		
Localization	ICA	164	3 (42.9%)	5.85 (1.25–27.04)	0.024
	Other	39	4 (57.1%)		
LTA > 50%	No	155	3 (42.9%)	4.41 (0.94–23.74)	0.061
	Yes	48	4 (57.1%)		
Arterial hypertension		115	5 (71.4%)	1.72 (0.34–11.92)	0.52
Diabetes mellitus		22	2 (28.6%)	3.91 (0.59–24.92)	0.16
Smoking		46	3 (42.9%)	2.74 (0.55–13.86)	0.21

## Data Availability

The data presented in this study are available on request from the first author. The data are not publicly available due to patient privacy protection.

## Abbreviations

The following abbreviations are used in this manuscript:

ADP	Adenosine diphosphate
APT	Antiplatelet therapy
ASA	Acetylsalicylic acid
CT	Computed tomography
DAPT	Dual antiplatelet therapy
EPV	Events per variable
FDS	Flow diversion stents
HC	Hemorrhagic complications
IA	Intracranial aneurysms
LTA	Light transmission aggregometry
MRI	Magnetic resonance imaging
mRS	Modified Rankin scale
NIH	National Institutes of Health
NOA	Novel oral anticoagulants
NSAIDs	Non-steroidal anti-inflammatory drugs
PED	Pipeline embolization device
PRU	P2Y12 reaction units
ROC	Receiver operating characteristic
SNIS	Society of NeuroInterventional Surgery
TEC/TECs:	Thromboembolic complications
VN	VerifyNow
